# Ten Years and Counting: Moving Leucine-Rich Repeat Kinase 2 Inhibitors to the Clinic

**DOI:** 10.1002/mds.26075

**Published:** 2014-12-01

**Authors:** Andrew B West

**Affiliations:** Center for Neurodegeneration and Experimental Therapeutics, Department of Neurology, University of Alabama at BirminghamBirmingham, Alabama, USA

**Keywords:** PARK8, neurodegeneration, neuroprotection, kinase inhibition

## Abstract

The burden that Parkinson's disease (PD) exacts on the population continues to increase year after year. Though refinement of symptomatic treatments continues at a reasonable pace, no accepted therapies are available to slow or prevent disease progression. The *leucine-rich repeat kinase 2* (*LRRK2*) gene was identified in PD genetic studies and offers new hope for novel therapeutic approaches. The evidence linking LRRK2 kinase activity to PD susceptibility is presented, as well as seminal discoveries relevant to the prosecution of LRRK2 kinase inhibition. Finally, suggestions are made for predictive preclinical modeling and successful first-in-human trials. © 2014 The Authors. *Movement* Disorders published by Wiley Periodicals, Inc. on behalf of International Parkinson and Movement Disorder Society.

The upcoming tenth anniversary of the discovery of mutations in the *leucine-rich repeat kinase 2* (*LRRK2*) gene in Parkinson's disease (PD) highlights numerous achievements in discovery and innovation (Fig. 1). In the face of many pharmaceutical companies slashing neuroscience research programs,^1^ combined with the shrinking National Institutes of Health budget,^2^ resources devoted to understanding LRRK2 in PD have managed to steadily increase. Indeed, there is compounding enthusiasm and optimism for what LRRK2 can divulge about the inner workings of PD. Optimism also exists in industry and academia alike for targeting the LRRK2 protein for therapeutic intervention in neurodegeneration. Yet, lessons from Huntington's disease and Alzheimer's disease show a long and convoluted road between gene discovery and drug development. Rational strategies that rely on accurate comprehension of pathobiological mechanisms are likely required to identify efficacious therapeutics. Focusing on the last 10 years of work, this perspective article provides a context for exploring the critical issues related to LRRK2 in PD susceptibility and therapeutic development. Specific suggestions for the advancement of LRRK2-targeting small-molecule kinase inhibitors to successful first-in-human studies are proposed.

**Figure 1:**
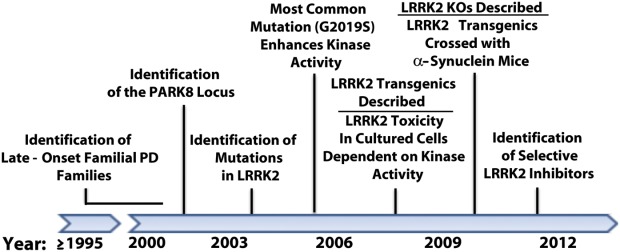
Timeline of key events for the development of LRRK2 therapies. [Color figure can be viewed in the online issue, which is available at http://wileyonlinelibrary.com.]

## Linking LRRK2 to PD

Initial excitement with *LRRK2* was not that another locus was found to be linked to another familial version of PD. In that case, *LRRK2*, localized to the *PARK8* locus, is promptly eighth in line and many more candidates having since followed (e.g., *PARK9-PARK20*). Rather, the first wave of excitement came from the descriptions of the families linked to the *PARK8* locus. Usually, familial PD-linked loci are confined to a few families, but *many* unique families across the globe were linked to *PARK8*.^3^–^5^ Not surprisingly, the discovery of mutations in the *LRRK2* gene in late 2004 was disclosed by several genetic groups collaborating into two independent articles published at the same time.^6^,^7^ Many other groups around the world soon followed with additional disclosures of *LRRK2* mutations.^8^–^10^

The second source of excitement was the nature of the disease linked to *PARK8*. Usually, familial parkinsonism involves early-onset forms of disease, often in concert with neurological symptoms not usually associated with late-onset typical disease. The importance of these Mendelian-inherited genes in idiopathic PD then becomes reliant on downstream pathological, functional, or therapeutic approaches. *PARK8* needs no additional studies to demonstrate importance in late-onset PD. One of the largest, best described families linked to *PARK8* was reported in 1995 by Ronald Pfeiffer and Zbigniew Wszolek who concluded that “This large kindred appears to represent a neurodegenerative disorder closely resembling, if not identical to, idiopathic PD.”^11^ This prescient observation has borne out in the last decade remarkably unscathed, even in the face of issues that commonly fog coherent genotype-phenotype linkages, such as clinic bias in subject ascertainment and publication bias of outlier families and cases.

**Figure 2:**
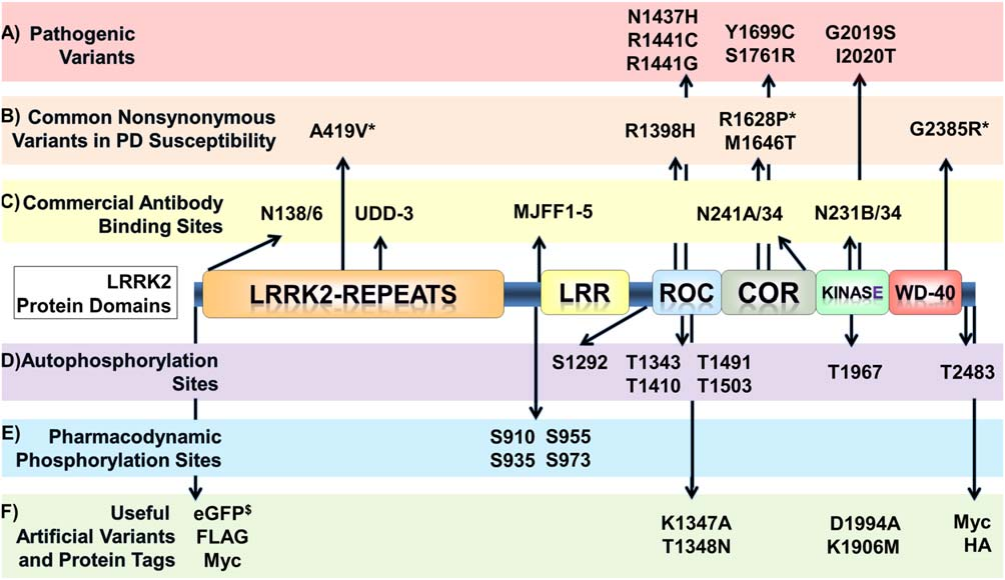
Selected variants and features in LRRK2 useful for the development of LRRK2-targeting therapies. Arrows reflect approximate position relative to conserved LRRK2 domains. (A) Pathogenic variants, proven by familial segregation, that cause late-onset PD. (B) Variants >1% frequency that are protective or disease-associated, * are variants in Asian populations. R1398H may be the functional variant in a protective haplotype with N551K. (C) Sensitive and specific commercial monoclonal Abs that can detect human and rodent LRRK2. Positions of binding are shown. (D) LRRK2 autophosphorylation sites proven with phospho-specific Abs. (E) Phosphorylation sites on the LRRK2 protein that are not autophosphorylation sites and do not measure LRRK2 activity, but effectively track LRRK2 kinase inhibition, and binding to 14-3-3 proteins. (F) Epitope tags and fluorescent proteins that can be appended to the N- or C-terminus of LRRK2 that have been shown, in biochemical assays, to retain LRRK2 kinase and/or GTPase activity. FLAG (acidic) and bulky proteins such as eGFP have not been compatible with active LRRK2 when attached to the C-terminus. ^$^eGFP, and many other fluorescent proteins, have been appended successfully to the N-terminus. Abbreviations for the LRRK2 protein domains include “LRRK2-repeats” that encode armadillo-like and ankryin-like repeats, “LRR” that is leucine-rich repeats, “ROC” that is ras-of-complex (i.e., GTPase), “COR” that is c-terminal of ras-of-complex, “kinase” that is the kinase domain, and “WD-40” that is WD-40-like repeats. eGFP, enhanced green fluorescent protein. [Color figure can be viewed in the online issue, which is available at http://wileyonlinelibrary.com.]

There are dozens of common nonsynonymous variants scattered throughout the *LRRK2* gene in various populations and individuals (http://www.uniprot.org/uniprot/Q5S007) and, possibly, hundreds of rare or idiosyncratic variants. Only a minority of these variants are linked to PD. As yet, there is no biochemical assay, no definitive molecular biology test, to conclusively demonstrate the pathogenicity of a particular variant. *Pathogenic* mutations in *LRRK2* (listed in Fig. 2A) are identified solely by their ability to segregate with disease in families. Idiosyncratic variants, no matter their identity or biochemical effects, cannot be interpreted as pathogenic without strong familial data that generally rely on DNA analysis from more than 5 affected subjects and at least as many unaffected subjects.

Although pathogenic variation in *LRRK2* is rare in humans, common genetic variants (e.g., minor allele frequencies of greater than 1% in a particular population) in the *LRRK2* gene are well established to affect susceptibility to disease. Some of these susceptibility variants are listed in Figure 2B. The largest whole genome-association study to date, involving 13,708 PD cases and 95,282 controls, places *LRRK2* among the top genes linked to PD susceptibility.^12^ In consideration of both familial and population studies, apart from *α-synuclein* (*PARK1/4*), no other gene shares as strong a relationship to late-onset PD.

In late-onset PD genetics, frequencies of pathogenic mutations are usually incredibly low in clinical populations, and associated presentation of the inherited disease in carriers is highly variable. The remarkably high frequencies of *LRRK2* mutations in late-onset PD have allowed unprecedented insight into *LRRK2*-linked phenotypes. Two salient features have emerged: First, there are no reliable clinical measures or tests to identify a *LRRK2* mutation carrier from idiopathic late-onset PD, short of genetic testing.^13^ In clinical populations, many *LRRK2* carriers fail to report a family history of disease and thus are understood as sporadic cases.^14^ This is owing, in part, to the second feature critical for understanding *LRRK2* in PD: Pathogenic mutations are not fully penetrant.

In Ashkenazi Jewish cohorts of PD, lifetime penetrance is estimated at less than 30% for developing PD.^15^,^16^ To put the *LRRK2* G2019S mutation in context with another genetic factor unambiguously linked to late-onset PD, mutations in the *GBA* gene show 9% overall penetrance for PD in Ashkenazi Jews.^17^ In the North African Berber cohorts, the lifetime penetrance appears to be much higher at 80%.^14^ Penetrance in typical Caucasian populations is not clear, but is the subject of scrutiny by http://23andme.com and other active consortia.^18^ Nevertheless, other factors besides *LRRK2* mutations are necessary for the development of PD.

## LRRK2 in the Kinome

Genetic studies have a habit of identifying proteins in neurodegenerative disease that make terrible targets for traditional therapeutic interventions. Of the 7,668 unique genes associated with known or potential druggability, frustratingly few of them are associated with PD.^19^ Indeed, many of the *PARK* loci highlight loss-of-function recessive forms of disease (e.g., *parkin*, *PINK1*, and *DJ-1*). Because most therapies in the clinic, particularly small-molecule based, tend to attenuate or ablate the activity of a protein target, restoring complex function that is lost can be much more challenging.

As a protein kinase and prominent card-bearing member of the druggable proteome, LRRK2, in many respects, is the most exciting drug target identified in modern PD research. Human DNA encodes 518 protein kinases, and this collection of protein, known as the kinome, is included in the druggable proteome. However, LRRK2 bears little resemblance to other protein kinases. LRRK2 is awkwardly nestled with other problematic proteins in the so-named “tyrosine-kinase-like family,” more by virtue of the nonspecific fact that LRRK2 is a multidomain protein versus anything known about function or expression profiles.^20^ Within superfamilies in the kinome, the encoded kinase domains are often so inbred in sequence similarity that it becomes difficult to find small molecules that interact with only one class of kinase or an individual protein. Specificity, not potency or other drug-like properties, is the first fundamental problem with targeting proteins such as LRRK2.

To tackle specificity issues, exploitation of LRRK2-specific sequences and structures in the adenosine triphosphate (ATP) pocket present a way to navigate through the usual quagmire of off-target interactions. LRRK2 harbors unusual amino acids in kinase subdomains that are otherwise highly conserved across the kinome. For example, the DFG hinge motif, critical to “in” versus “out” conformations in kinase activation, is DYG in LRRK2. The LRRK2 DYG motif is further altered with the pathogenic LRRK2 mutation, G2019S, to a DYS motif. These LRRK2-specific sequences encode the very amino acids that form the ATP pocket that many kinase inhibitors interact with for therapeutic gain.^21^

Besides unique kinase-domain sequences, there are other features specific to LRRK2. LRRK2 is regrettably named because several other human protein kinases have leucine-rich repeat domains (e.g., leucine-rich repeat receptor kinases), but no other protein has a tandem encoded GTPase domain with proven enzymatic function. This defining enzymatic duet is conserved across >500 million years of evolution between humans to single-celled organisms such as *Dictyostelium*,^22^ demonstrating obvious essentiality to the arrangement. As with the LRRK2 kinase domain, the LRRK2 GTPase domain also diverges from other G-protein families (guanine nucleotide-binding proteins).^23^

Given that the LRRK2 GTPase domain cannot reasonably be assigned to any of the main G-protein families (e.g., Ras, Rho, Rab, Arf, or Ran), a new family called “Ras-like proteins in complex with other domains” (ROC) was created. In humans, the family is comprised of LRRK1 and LRRK2 and substantiates the overall evolutionary distance of LRRK2 from other well-characterized kinases. Alignments of the most intrinsically conserved LRRK2 GTPase residues against the prototypical H-Ras protein suggest that the amino acids commonly used in biochemical studies to inactivate GTPases (e.g., H-Ras sequence glycine 12 and glutamine 61) are already substituted in the LRRK2 GTPase domain. Furthermore, the typical phenylalanine-to-leucine mutation, useful for studying many G proteins (e.g., position 28 in H-Ras), is also natively a leucine in LRRK2. These two features are indicative of low affinity for nucleotides, compared to other G proteins, and a mostly inactive enzyme in cells. The notion that the vast majority of the LRRK2 enzyme lay enzymatically dormant in cells has, to the chagrin of LRRK2 biologists, largely borne out experimentally. Nevertheless, a recent first study of its kind suggests therapeutic potential for molecules that may bind to the GTPase domain.^24^

The uniqueness of the LRRK2 enzyme thus presents a gift and a curse: a gift in that there are viable protein domains and interactions that are unique to LRRK2, so molecules should exist that interact only with LRRK2 and therefore subvert off-target interactions. The curse is that decades of research on how G proteins regulate protein kinases may not provide relevant insight into LRRK2 function, given that the LRRK2 enzyme diverged quite early from other better-characterized kinases and G proteins.

## Impact of Pathogenic Mutations on LRRK2 Activity

In order to therapeutically target LRRK2, it would be useful to understand the effects of pathogenic mutations on LRRK2 function. LRRK2 may have dozens of different activities in hundreds of different kinds of cells, so narrowing down the property most clearly linked to PD would provide a reasonable foundation to pursue and validate targeted therapeutics. Less than 1 year after the discovery of mutations in *LRRK2*, it was possible to clone *LRRK2*, develop reasonable polyclonal antibodies (Abs), and create an initial assay to measure LRRK2 kinase activity.^25^ During this time, some reports, based on homologous modifications made to other protein kinases, suggested that PD mutations would inhibit kinase function.^26^ In contrast, the first actual assay demonstrated an activating effect for the R1441C and G2019S with respect to LRRK2 autophosphorylation.^25^

**Figure 3:**
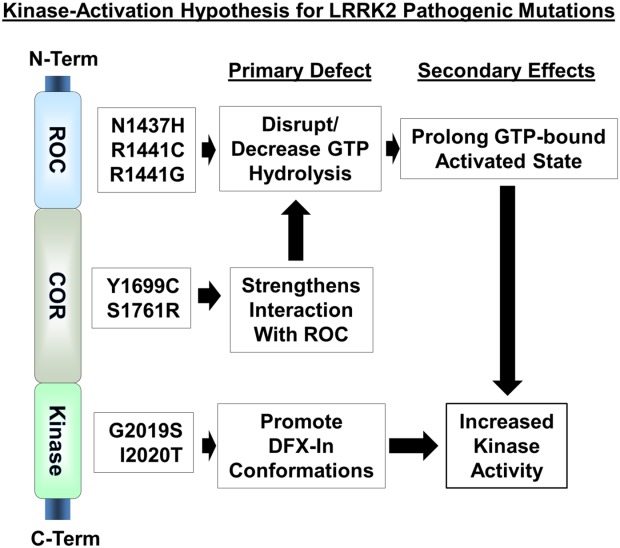
Summary of a kinase-activation hypothesis for the mechanism of action of LRRK2 pathogenic mutations. GTP, guanosine triphosphate. [Color figure can be viewed in the online issue, which is available at http://wileyonlinelibrary.com.]

Kinase-activating effects of *LRRK2* mutations could be caused by many factors. Based on the distribution of pathogenic mutations across the LRRK2 ROC, COR, and kinase domain (Fig. 2A), it is not surprising that different mutations have been postulated to affect kinase activity in different ways (Fig. 3). The most common *LRRK2* mutation, G2019S, up-regulates kinase activity in a fundamental way that is revealed through every (published) assay. However, there are no other pathogenic *LRRK2* mutations that enjoy this relationship. In some experimental settings, pathogenic *LRRK2* mutations, such as R1441C, Y1699C, and I2020T, fail to distinguish LRRK2 kinase-associated activities from wild-type (WT) baselines, whereas, in other experiments, the mutations up-regulate kinase activities. Many of the initial controversies can be explained through a more detailed dissection of kinase activation and kinetics. Part of the problem is that experimental paradigms that describe LRRK2 kinase activity often rely on underlying assumptions that may confound interpretation when evaluating the effect of pathogenic mutations.

First and foremost may be the assumption that a representative proportion of the active state of the LRRK2 enzyme is captured and preserved from protein purified from tissues or cells for use in a particular assay. For example, the I2020T alters the proportion of protein in an active DYG-in pocket conformation, but the increase in kinase activity can be negated depending on assay conditions and the nature of the kinase substrate.^27^ Similar active-state stabilization mechanisms may be occurring in the ROC domain for other pathogenic mutations.^28^ Active-state conformations can also be affected variably by at least seven other known factors, usually present in unknown stoichiometries with respect to enzyme, in published kinase-assay experiments: (1) LRRK2 protein cofactors and interactors such as 14-3-3 and heat-shock proteins that coelute with LRRK2, as well as other less-abundant interacting factors such as ArfGap1, (2 and 3) metal such as Mg^++^ bound to the GTPase domain, and kinase domain, (4) guanine nucleotide bound to the GTPase domain, (5) adenosine nucleotide bound to the kinase domain, (6) peptide substrate docked in the kinase domain, and (7) substrates of autophosphorylation docked to the kinase domain (readily saturable). All seven of these substrates or cofactors can, in a coordinated way, interact to affect LRRK2 kinase output, either directly or indirectly. Establishing a credible baseline *in vitro* for all of these factors for a relevant comparative analysis becomes difficult. Emphasis has therefore been placed on assays capable of measuring LRRK2 kinase activities directly in cells that express LRRK2.

Despite tremendous efforts to identify protein phosphorylation events dependent on LRRK2 activity in cells, there are no known accepted substrates of LRRK2 kinase activity. However, there is one standing exception: LRRK2 itself. Evaluation of LRRK2 *cis-*phosphorylation (i.e., autophos) has revealed a number of surprises. The first mass spectrometry (MS) analysis to map autophos residues in LRRK2 identified an N-terminal cluster of phospho-serines (e.g., pS910 and pS935), but these were also detected in kinase-dead LRRK2 protein.^29^ The next wave of studies, using LRRK2 protein subjected to in vitro kinase assays before analysis, successfully detected a slurry of autophos residues not concentrated in the kinase domain as anticipated, but in the GTPase domain (Fig. 2D).^30^–^32^ Seven sites have been confirmed with phospho-specific Abs (Fig. 2D). However, when most autophos-Abs are applied to tissue or cell lysates containing endogenous LRRK2, no significant signal can be resolved.^32^,^33^ Either the autophos sites are so low in abundance in cells, for example, because LRRK2 is mostly inactive, or the sites themselves are exclusive products of in vitro conditions.

A subsequent quantitative MS study provided evidence that the autophos pS1292 residue is particularly abundant and therefore be detected directly from lysates.^34^ It is conceivable that Abs to pS1292 may be used to affinity purify LRRK2 protein in strategies to enrich and enable the detection of less-abundant autophos sites. The Abs specific to pS1292 have been hypothesized as a more direct and relevant way to determine LRRK2 kinase activity,^34^ with the caveat that any one particular substrate may not predict activity toward other substrates.

In the first study directly measuring LRRK2 autophos activity from LRRK2 protein expressed in cell lines, pathogenic LRRK2 mutations increased the proportion of pS1292-LRRK2, relative to total LRRK2.^34^ With the exception of the Y1699C mutation, this study mimicked previous studies that measured overall autophos incorporation in kinase assays using radioactive adenosine nucleotide.^29^ In combining GTPase pathogenic mutations in *cis* with kinase-domain mutations, the activating effects on kinase function become quite dramatic, again mimicking some earlier studies measuring total phosphate incorporation.^32^ Still, outside of measuring autophos in cells, the activating effects of individual mutations outside of the kinase domain have variable or negligible effects on some aspects of kinetics in certain experimental paradigms. Though the identification of bone-fide LRRK2 kinase substrates important in LRRK2-linked cellular pathways might overshadow studies that measure the effects of pathogenic mutations through measuring autophos levels, for now, the results closest to cellular (and thus relevant) conditions support a kinase-activation hypothesis for LRRK2-linked pathogenesis (Fig. 3).

## Small-Molecule LRRK2 Inhibitors

There is no longer debate as to whether small-molecule kinase inhibitors can be highly selective and clinically efficacious.^35^ Tremendous efforts have focused on the identification of small-molecule kinase inhibitors that selectively target LRRK2 kinase activity to bring enzymatic function back to normal (e.g., WT), or ablate activity altogether, in the hopes of a neuroprotection strategy for PD. Initial studies identified several classes of nonselective kinase inhibitors (e.g., molecules that inhibit >20 known kinases at 50% inhibitory concentration <1 µM) with excellent (low nanomolar) potency against LRRK2. These molecules include staurosporine, sunitinib, CZC 54252, and TAE684. Owing to their promiscuity, these compounds have limited utility when applied to cells and cannot provide information on the safety of selective LRRK2 inhibition.

More recently, molecules with improved selectivity have been described on several distinct scaffolds. The first of these, aptly dubbed LRRK2-IN-1, has been widely deployed in numerous high-profile biological studies that attempt to define the role of LRRK2 in model systems and/or rescue pathological effects of G2019S-LRRK2.^36^ However, LRRK2-IN-1, as pointed out in the original description, also inhibits ubiquitous and critical enzymes, such as Erk5, with near equal potency, such that it is difficult or impossible to discern LRRK2 function in most cellular systems. In addition, LRRK2-IN-1 does not cross the blood–brain barrier (BBB) and has numerous undesirable pharmacokinetic problems not easily remediated.

Two other LRRK2 inhibitor scaffolds were identified with improved specificity, pharmacokinetics, and distribution in vivo. GSK2578215A is a highly selective compound with minimal inhibition of other kinases, of 460 kinases tested, and shows evidence of brain penetration.^37^ Yet, GSK2578215A fails to inhibit LRRK2 enzyme in the brain, presumably because of poor free drug availability. Based loosely on the LRRK2-IN-1 scaffold, the inhibitor, HG-10-102-01, shows promising selectivity, can cross the BBB, but still bears suboptimal pharmacokinetics, permeability, stability, and other in vivo attributes that otherwise preclude use of this molecule in vivo.^34^ Optimizations of the HG-10-102-01 series led to the development of GNE-0877 and GNE-9605.^38^ These compounds may be suitable for some in vivo applications, but the selectivity profiles of these compounds are less than ideal.

Despite few options currently available for in vivo experiments, the limiting factor is not the ability to resolve efficacy. Abs directed to any of the phosphorylated residues identified in LRRK2 would theoretically track LRRK2 kinase inhibition, including the phospho-serine Abs that are not autophosphorylation sites.^36^ But, in practice, it appears that the only autophos site that might be abundant enough to detect with routine methods (e.g., western blot) might be pS1292.^34^ Abundant N-terminal phosphorylation sites, such as pS935 and pS910, that do not directly measure LRRK2 activity, but faithfully correlate with LRRK2 inhibition, have proven useful in dozens of studies. However, these sites are not preferred over autophos sites because they would also track inhibition of other kinases (i.e., not LRRK2) that phosphorylate the LRRK2 sites. In addition, 14-3-3 proteins require pS935 and pS910 phosphorylation to bind to LRRK2, so factors that alter 14-3-3 function in cells may have indirect effects on pS935 and pS910 phosphorylation by allowing other kinases to interact with the sites that would normally be blocked by 14-3-3 protein bound to LRRK2.

Because of patent-life vulnerability, it is reasonable to expect that the best LRRK2 inhibitor series currently remain undisclosed. Published inhibitor series likely harbor critical flaws that preclude consideration as strong clinical candidate molecules. Nevertheless, the inhibitor series that have been publicized show relatively good selectivity and potency toward LRRK2, so that better molecules should exist within the scope of reasonable amounts of effort.

## Preclinical Approaches for the Identification of Efficacious LRRK2 Kinase Inhibitors

In PD research, there are no known neuroprotective treatments, so identification of a model system that predicts clinical success for neuroprotection cannot exist with certainty. However, several major advances in PD research that preceded the discovery of *LRRK2* in PD by a few years have had resounding trickle-down effects in preclinical approaches that should be considered in testing LRRK2 kinase inhibitors. First, Abs directed to abnormal *α*-synuclein, applied in a systematic manner to postmortem archived brain sections from PD, led to the identification of a staging system that followed a common progression for *α*-synuclein lesions.^39^ Together with familial genetic studies,^40^ as well as genome-wide association studies,^12^ there is little uncertainty that *α*-synuclein is the single most important genetic and pathological factor in PD progression and susceptibility. Modeling *LRRK2* in the pathobiology of PD would necessarily involve aberrant *α*-synuclein if the hope is to predict the effects of LRRK2 inhibition in mechanisms relevant to PD.

Most in vitro studies have concluded that the various toxicities caused by overexpression of LRRK2 are dependent on LRRK2 kinase activity.^41^–^43^ Though there is limited evidence that LRRK2 itself is overexpressed in PD, most cells in culture appear to have little or no LRRK2 protein that can be detected,^44^ so overexpressing the protein is required, in many cases, to study LRRK2 function. However, aberrant overexpression of LRRK2, particularly in an acute and transient manner, runs the clear risk of deregulating the enzyme so that interactions that would not normally take place become much more likely.

Another salient, but also highly variable, result of overexpression of LRRK2 in neurons and cell lines alike is the development of skein-like LRRK2 aggregates.^45^–^47^ These features have never been observed in cells in the brain of even LRRK2 transgenic (Tg) animals that overexpress LRRK2 many fold above endogenous levels, much less observed in normal rodent brain or human healthy and PD brain.^48^–^50^ Yet, many studies have prioritized observations involving LRRK2 aggregation resulting from overexpression. Other studies have focused on induced pluripotent stem cells (iPSCs) to understand LRRK2. Because *LRRK2* may be localized to an unstable segment of chromosome 12p13 that is subject to copy number instability in cell lines,^51^ individual iPSC clones derived from fibroblast sources may show strong heterogeneity with respect to the *LRRK2* gene locus and should be evaluated closely for instability.

There are no consensus assays emergent from the literature that have been easily reproduced across laboratories to assess LRRK2 toxicity. LRRK2 function, relevant to mechanisms important in PD, may be alternatively understood in combination with dysfunction elicited by other factors underlying PD. In the first large-scale study involving Tg mice that conditionally overexpress mutant (A53T) *α*-synuclein, deletion of the LRRK2 gene was found to provide protection from broad-sweeping damage to the forebrain.^52^ In these strains of mice, overexpression of *α*-synuclein in CamKII-positive cells caused fragmented Golgi and elicited neuritic retraction in neuronal subpopulations at the 12-month time frame. However, on a LRRK2 knockout (KO) background, there was less damage to neurons and decreases in associated Iba-1 microglial reactivity. Concomitant overexpression of G2019S-LRRK2 hastened neuronal damage caused by A53T *α*-synuclein and enhanced neuroinflammation and associated degeneration. Inherent variabilities and complicated genetic crosses suggest this Tg system may not be the most straightforward way to identify efficacious LRRK2-targeting molecules. Also, other studies show that LRRK2 KO or overexpression did not affect A53T *α*-synuclein phenotypes, such as premature death.^53^,^54^

More recent studies show that it may be possible to compress PD-relevant LRRK2-dependent phenotypes into a much shorter time frame. Direct delivery of *α*-synuclein to the SN through viral vectors causes dopaminergic neurodegeneration, and this process also models proinflammatory processes important for cell loss.^55^,^56^ At the 4-week time point post-rAAV2-*α*-synuclein delivery in rats, LRRK2 KO animals were broadly protected from dopaminergic neurodegeneration.^57^ The acute nature of this model may provide a reliable platform to evaluate LRRK2 small-molecule inhibitors, should pharmacological inhibition faithfully mimic effects observed in KO animals. Under pathological stimuli, LRRK2 expression becomes induced in proinflammatory brain myeloid cells, and both in isolated (cultured) myeloid cells and myeloid cells in the brain, LRRK2 knockdown attenuates proinflammatory responses from these cells.^57^,^58^ Lipopolysaccharide exposures that cause neurodegeneration may also represent viable model systems to resolve LRRK2 kinase inhibitor efficacy for reducing proinflammatory responses.

The effects of chronic inhibition of LRRK2 should be closely evaluated in preclinical models to identify issues that will have to be addressed in clinical trials. There have been no described phenotypes associated with heterozygous knockdown of LRRK2 in mice or rats. Homozygous LRRK2 KO animals might mimic the effects of a perfect drug that achieves complete ablation of the target at all times. However, protein kinases can also serve in important functions that are independent of kinase activity. For example, KO of the CamKII protein impairs presynaptic plasticity and vesicle docking at the synapse, whereas kinase inhibition of CamKII does not impair these functions.^59^ Nevertheless, in LRRK2 KO rodents, three changes in particular have been highlighted through relatively exhaustive studies that may be of particular concern: kidney and lung pathology as well as immunological homeostasis changes.^53^,^60^ Whereas LRRK2 KO rodents do not show strong (or any) phenotypic evidence of kidney or lung failure, there is reproducible tissue and cellular abnormalities in these organs. Immune system changes, namely, the total numbers of some types of circulating cells, are subtle, but also reproducible. Future pharmacotoxicity studies involving LRRK2 clinical candidate inhibitors will prioritize comparisons of dosages and timelines against toxicities associated with these tissues and cells.

## First-in-Human LRRK2 Inhibitor Trials

Although there are no guarantees for the success of individual LRRK2 inhibitors, there are no data either that would suggest eminent failure for LRRK2 inhibitors in safety trials. A solid foundation of preclinical studies will help elucidate the type of benefit a subject participating in an efficacy trial might expect. Without a good base of knowledge, incorrect assignment of endpoints can result in the failure of an otherwise viable therapeutic. If LRRK2 critically modifies initial progression of motoric deficits in PD, for example, by promoting survival of dopaminergic neurons, a primary endpoint could be used, such as measuring the time interval from diagnosis of PD to the requirement of dopaminergic therapy. Meaningful changes in clinical scales, such as MDS-UPDRS, could also be used, although subjective metrics embedded within most clinical scales reduce power. Other, more quantitative endpoints may be possible. If LRRK2 inhibitors are predicted to reduce inflammation in the brain in various stages of disease, one endpoint may be reduction of signal elicited by a PET ligand that measures neuroinflammatory responses. Likewise, reductions in correlated serum or cerebral spinal fluid inflammation markers may resolve therapeutic efficacy. Clearly, additional preclinical studies are critical to refine endpoints in animal models that could be recapitulated, with some semblance, into an efficacy study in humans.

Large cohorts of LRRK2 mutation carriers with and without PD have already been assembled by the Michael J. Fox Foundation and others, in part to facilitate future clinical trials for LRRK2 inhibitors. The challenges in designing efficacy studies with healthy mutation carriers are that age at onset can be highly variable, particularly in Ashkenazi Jewish populations. In efficacy studies involving LRRK2 mutation carriers with PD, it would have to be assumed that benefit can be derived at various stages of disease. However, there are no studies yet that have made the point to evaluate LRRK2 inhibition during disease progression in preclinical models. Unfortunately, these types of studies are often more challenging to design than initial studies and are not particularly prioritized by many funding agencies. However, oft-neglected meticulous preclinical modeling studies could be required for successful trial design.

Involvement of idiopathic late-onset PD cases in LRRK2 inhibitor efficacy trials would lend toward easier recruitment and no need for genetic counseling, compared to a trial involving LRRK2 mutation carriers. This type of trial would presume a broad role for LRRK2 in the progression of PD. Clearly, research efforts will need to be intensified on appropriately powered preclinical studies that integrate LRRK2 compounds, as they become available, together with genetically modified rodents.

An endpoint omitted in many (if not most) efficacy trials in PD is validation in subjects that the drug has achieved the desired on-target effect. This is a systemic problem in biomedical research and has resulted in billions of dollars wasted.^61^ To test the hypothesis that LRRK2 inhibitors might offer benefit to patients with PD or individuals at high risk for PD (e.g., G2019S-LRRK2 carriers), mere common sense dictates that verification of LRRK2 inhibition should be determined for individual subjects undergoing treatment. An adaptive clinical trial design may be particularly useful in this regard to emphasize the importance of on-target action. However, though access to the LRRK2 enzyme in brain tissue is easily obtainable in preclinical models, there is no such direct availability in human subjects participating in a clinical trial. Two new approaches show promise for tracking LRRK2 kinase activity during a clinical trial.

LRRK2 is encapsulated in microvesicles called exosomes that are in circulation in human cerebral spinal fluid and urine.^62^ Exosomes house thousands of proteins derived directly from their parental cell cytosol.^63^ Release of LRRK2 in exosomes is partially dependent on kinase activity and 14-3-3 binding, so that in subjects treated with LRRK2 inhibitors, both phospho-LRRK2 as well as total LRRK2 would be expected to be reduced.^62^ Given the interindividual variability of LRRK2 expression in extracellular fluids, baseline measurements would be required to resolve differences caused by LRRK2 inhibitors.

A second potentially complementary noninvasive approach to track LRRK2 inhibition may be application of PET ligands specific for the LRRK2 ATP-binding pocket (e.g., a LRRK2 small-molecule kinase inhibitor). Many clinically approved kinase inhibitors show excellent potencies with effective inhibitory concentrations that titrate against active enzyme concentrations in cells. A slightly lower-affinity LRRK2 small-molecule inhibitor with rapid turnover could be implemented as a PET ligand. For example, the HG-10-102-01 series of inhibitors naturally clears rapidly from the brain and would fail to bind to LRRK2 should the pocket be already occupied by a more potent class of inhibitor.

## Concluding Remarks

Around the time of the publication of the pair of genetic studies linking *LRRK2* to PD, an economic report predicted that the cost for the development of a new therapeutic would cost over $1 billion USD over a 10- to 15-year period, from preclinical work through phase clinical trial.^64^ But PD fits poorly into this optimistic mold. PD is not an infectious disease that can be tracked and modeled directly, and it is not a cancer with tumors that can be measured, probed, and dissected. PD has no accepted treatments that slow or otherwise modify progression. As such, there are no animal models that provide solid predictive validity that would otherwise expedite novel neuroprotective treatments.

Because of all that is unknown and therefore risky, a billion dollars for a neuroprotective treatment in PD would be a bargain given that the annual cost of PD for the economy exceeds $14 billion.^65^ The economic return for something effective, even in the short term, would vastly outweigh any conceivable development costs for an individual therapy. Ten years for development of the first neuroprotective drug, from bench to bedside, would be miraculous. This will not be the case for LRRK2 inhibitors. The question becomes whether LRRK2 is the right target to dedicate extremely limited resources in the hopes of the first neuroprotective therapy in PD.

Based on the last 10 years of research, the case for targeting LRRK2 kinase activity in carriers of the G2019S mutation is as strong as it gets. For LRRK2 noncarriers with PD (e.g., the vast majority of PD cases), ultimately a properly designed clinical trial with the right inhibitor will be required to understand the true role of LRRK2 in the pathobiology of PD. Some 50 years ago, the path forward that led to the last major advance in PD therapeutics, getting dopamine back into the brain, was a bumpy path, but brightly lit by solid rationale and available pharmaceuticals. The path forward for LRRK2 inhibitors seems just as brightly lit. We are certainly overdue for the next major advance in PD therapeutics.
